# Advancements in Sensor Fusion for Underwater SLAM: A Review on Enhanced Navigation and Environmental Perception

**DOI:** 10.3390/s24237490

**Published:** 2024-11-24

**Authors:** Fomekong Fomekong Rachel Merveille, Baozhu Jia, Zhizun Xu, Bissih Fred

**Affiliations:** 1 School of Naval Architecture and Maritime, Guangdong Ocean University, Zhanjiang 524000, China; marvelous@stu.gdou.edu.cn; 2 School of Engineering, Newcastle University, Newcastle upon Tyne NE1 7RU, UK; zhizun@gdou.edu.cn; 3 College of Fisheries, Guangdong Ocean University, Zhanjiang 524088, China; 1252201246@stu.gdou.edu.cn

**Keywords:** underwater simultaneous localization and mapping (SLAM), sensor fusion, unmanned underwater vehicles (UUVs), Kalman filter, particle filter, graph-based SLAM, quantum sensors, AI-driven filtering, deep learning

## Abstract

Underwater simultaneous localization and mapping (SLAM) has significant challenges due to the complexities of underwater environments, marked by limited visibility, variable conditions, and restricted global positioning system (GPS) availability. This study provides a comprehensive analysis of sensor fusion techniques in underwater SLAM, highlighting the amalgamation of proprioceptive and exteroceptive sensors to improve UUV navigational accuracy and system resilience. Essential sensor applications, including inertial measurement units (IMUs), Doppler velocity logs (DVLs), cameras, sonar, and LiDAR (light detection and ranging), are examined for their contributions to navigation and perception. Fusion methodologies, such as Kalman filters, particle filters, and graph-based SLAM, are evaluated for their benefits, limitations, and computational demands. Additionally, innovative technologies like quantum sensors and AI-driven filtering techniques are examined for their potential to enhance SLAM precision and adaptability. Case studies demonstrate practical applications, analyzing the compromises between accuracy, computational requirements, and adaptability to environmental changes. This paper proceeds to emphasize future directions, stressing the need for advanced filtering and machine learning to address sensor drift, noise, and environmental unpredictability, hence improving autonomous underwater navigation through reliable sensor fusion.

## 1. Introduction

The oceans encompass over 71% of the Earth’s surface, serving a vital function in scientific, environmental, and industrial domains, including oceanographic research, marine ecosystem surveillance, and subsea infrastructure evaluation [1,2,3,4,5,6]. The intricate characteristics of underwater settings pose distinct problems for UUVs [7,8], especially in attaining dependable navigation and mapping. In contrast to terrestrial surroundings, underwater settings exhibit low visibility, swift signal degradation, and a lack of GPS, all of which impede precise localization and mapping [9,10]. Light and sound, vital for vision-based and sonar systems, deteriorate markedly due to water dispersion, absorption, and reflection, leading to degraded sensor data. Moreover, dynamic environmental factors—such as fluctuating currents, changing salinity, and turbidity—complicate the operational challenges for UUVs [11,12].

Recent research has increasingly concentrated on sensor fusion to address these problems, integrating data from several sensor modalities to improve the resilience and accuracy of SLAM systems [13,14,15,16]. Sensor fusion facilitates the amalgamation of proprioceptive sensors, such as inertial measurement units (IMUs) that convey data regarding the UUV’s internal condition, with exteroceptive sensors, including sonar, cameras, and LiDAR, which acquire environmental information for mapping and object recognition [17,18,19]. This amalgamation of sensors mitigates the limitations of individual sensors, resulting in enhanced precision and dependability in navigation.

This paper conducts a thorough evaluation of current research on sensor fusion in underwater SLAM, chosen for its relevance, novelty, and methodological rigor [17,20,21,22]. Emphasis is placed on research concerning noise, drift, and sensor degradation in underwater environments, particularly focusing on current advancements in deep learning and AI-based sensor fusion methodologies [23,24,25,26,27]. This review emphasizes contemporary difficulties and progress, offering insights into the strengths and weaknesses of current research while proposing avenues for improving UUV navigation in intricate underwater settings.

Notwithstanding significant advancements, existing SLAM systems encounter considerable obstacles in dynamic, GPS-denied settings characterized by restricted visibility and frequent signal loss [28,29,30]. Contemporary research frequently focuses on individual sensor modalities, such as compass, Doppler Velocity Logs (DVL), sonar, LiDAR, or visual SLAM, each with intrinsic constraints. For example, vision-based SLAM has difficulties in murky seas, sonar is limited by resolution, and IMUs are susceptible to drift over time [31,32,33]. Although certain sensor fusion techniques are integrated, these systems frequently struggle to achieve a balance between computing burden and real-time accuracy, particularly in intricate underwater environments characterized by fluctuating turbidity and illumination [8,34,35,36,37,38].

Conventional fusion methods, such as Kalman filters and particle filters, while efficient, face scalability challenges when including numerous sensors in nonlinear and unpredictable underwater environments [39,40,41]. This study delineates three primary research gaps: (1) insufficient real-time sensor fusion in dynamic, turbid environments; (2) computational difficulties in integrating multiple sensor types under complex underwater conditions; and (3) the necessity for sophisticated filtering techniques and machine learning models to improve real-time fusion by alleviating sensor drift, noise, and environmental variability [27,42].

This paper presents a fresh and comprehensive taxonomy of sensor fusion algorithms, categorized by mathematical principles, underwater [43] efficacy, and computing trade-offs. This categorization offers a sophisticated framework for assessing sensor fusion algorithms designed for underwater applications. This review contrasts previous research by examining established methods like Kalman and particle filters while also exploring novel breakthroughs in AI-driven and quantum sensor fusion techniques.

This work offers a comparative review of sensor fusion methodologies, including Kalman filters, Extended Kalman filters [44], particle filters [45,46], and graph-based SLAM [47,48], emphasizing their efficacy in mitigating difficulties such as drift, signal degradation, and noise. We evaluate contemporary multimodal sensor fusion techniques that include IMUs, Doppler velocity logs, sonar, cameras, and LiDAR, providing improved resilience and precision in practical applications. Innovations in quantum sensing and artificial intelligence (AI)-driven [49,50,51,52,53] adaptation filtering, including reinforcement learning and deep learning, are examined as potential transformative solutions for real-time adaptability in underwater environments.

The organized study on underwater SLAM with sensor fusion approaches, advanced computational frameworks, and new technologies like quantum sensors and AI describes current and future underwater navigation advances. The Introduction discusses underwater obstacles like low visibility and no GPS and how sensor fusion improves SLAM accuracy. Proprioceptive and exteroceptive sensors are crucial to underwater SLAM, as discussed in Section 2. Section 3 discusses the pros and cons of multiple sensor integration in SLAM odometry. Section 4 compares Kalman filters, particle filters, and graph-based SLAM for sensor fusion, including computing requirements, accuracy, and optimal applications. Section 5 examines computational efficiency, optimizing processing speed and memory for real-time underwater SLAM. In Section 6, case studies present experimental outcomes from military, industrial, and environmental studies. Future directions include hybrid quantum–AI models, deep learning, and computational optimizations for resilient, adaptable SLAMs in complicated underwater environments. The Conclusion concludes that sensor fusion and AI-driven SLAM technology can transform UUV underwater exploration.

The following section discusses SLAM sensors and how proprioceptive and exteroceptive technologies enable navigation and mapping in complicated aquatic settings to handle underwater issues.

## 2. Essential Underwater Sensors and Their Function in Facilitating Improved SLAM Systems

Underwater sensors are specialized instruments designed to monitor various physical and environmental characteristics, enabling effective data collecting and navigation in intricate and dynamic underwater environments. These sensors are essential for underwater exploration, facilitating autonomous underwater vehicles (AUVs) and remotely operated vehicles (ROVs) to collect real-time data for navigation and mapping. The underwater sensor systems are classified into proprioceptive sensors and exteroceptive sensors, as detailed in the subsequent section.

### 2.1. Proprioceptive Sensors

Proprioceptive sensors like accelerometers [54], gyroscopes, and magnetometers measure location, velocity, acceleration, and rotation to reveal a system’s internal state and motion. These sensors include accelerometers, gyroscopes, magnetometers, and others, all of which play vital roles in UUV navigation and control systems. Underwater vehicles require depth sensors, such as barometers, for navigation and measurement purposes. Barometers, a category of pressure sensors, gauge depth by assessing the pressure imposed by the water column above the sensor. Precise depth measurements are essential for sustaining the intended altitude during underwater operations.

DVL utilizes acoustic measurements to ascertain a UUV velocity concerning the seafloor. They are essential for dead reckoning, supplying velocity and positional data when GPS or alternative navigation signals are inaccessible, as in deep-sea exploration. Inertial measurement units (IMUs) [55,56,57,58,59] combine accelerometers, gyroscopes, and magnetometers to measure linear acceleration, angular velocity, and orientation. These sensors provide continuous feedback on the AUV’s movement, making them indispensable for maintaining precise navigation and orientation in challenging environments. IMUs have been extensively employed in aircraft navigation, mobile devices, and underwater robotics due to their ability to operate independently of external signals.

Acoustic Doppler current profilers (ADCPs) measure water current velocities using the Doppler effect. This information is essential for correcting navigation errors caused by underwater currents and maintaining precise control of UUV motion. Inclinometer sensors measure tilt or inclination angles, allowing UUVs to maintain balance and control in uneven or rugged underwater terrain. Thermocouples used for temperature measurement play a crucial role in environmental monitoring, helping AUVs navigate through thermoclines or temperature gradients in the water column.

**Figure 1:**
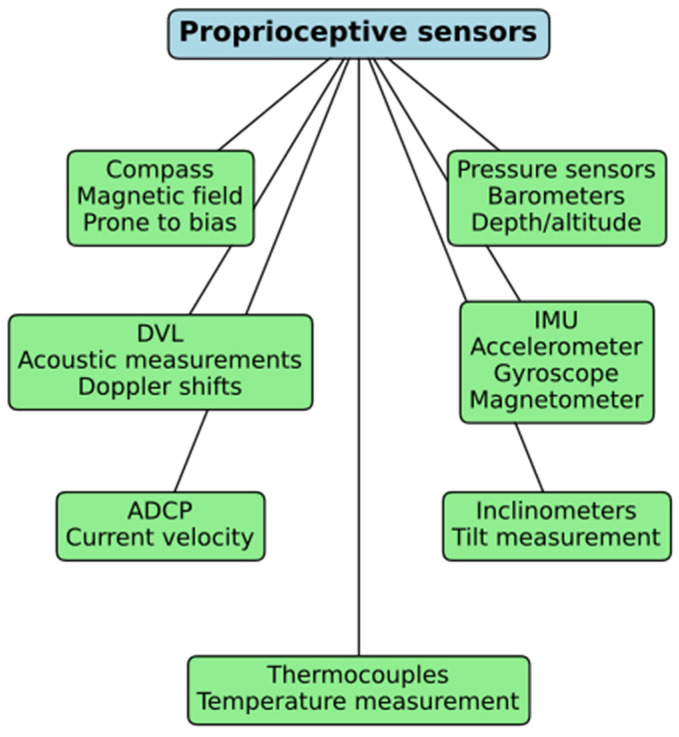
Proprioceptive sensors for aquatic navigation.

Figure 1 depicts the standard proprioceptive sensors utilized for underwater navigation. The sensors comprise compasses (susceptible to magnetic field interference); barometers (for depth assessment); DVLs for acoustic velocity evaluation; IMUs integrating accelerometers, gyroscopes, and magnetometers; acoustic Doppler current profilers (ADCPs for current velocity measurement); inclinometers (for tilt detection); and thermocouples (for temperature assessment). Every sensor enhances efficient underwater navigation and environmental awareness.

### 2.2. Exteroceptive Sensors

Exteroceptive sensors gather information from the environment, enabling UUVs to sense and map their surroundings. These sensors include cameras, sonar, and LiDAR, each contributing unique data points that help build a comprehensive understanding of the underwater world.

**Figure 2:**
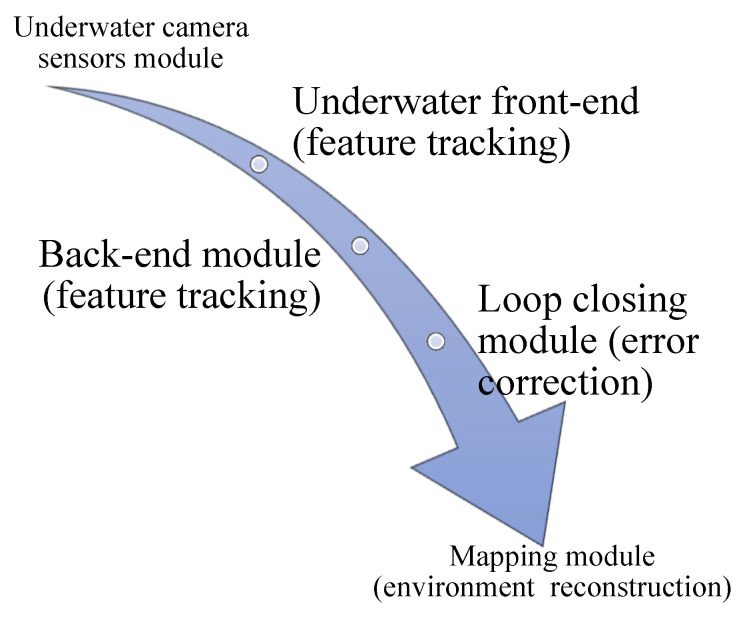
The typical architecture of a visual SLAM system.

Figure 2 provides a visual depiction of an underwater SLAM system architecture, illustrating the sequential modules involved in feature tracking and environmental reconstruction. The system commences with the Underwater Camera Sensors Module, succeeded by the Underwater Front-End for feature tracking. The Back-End Module facilitates supplementary feature tracking, while the Loop Closing Module enables mistake correction. The procedure concludes with the Mapping Module, which is tasked with environment reconstruction, facilitating precise underwater navigation.

Visual SLAM uses numerous camera types [60,61] to perceive the surroundings. Monocular cameras [62,63] compute 3D structures from 2D input and set initial distances depending on relative motion [64].

Parallax depth calculations allow stereo cameras [65,66] to localize underwater accurately. They use novel topological representations for real-time processing [67].

RGB-D depth cameras estimate distances using structured light or time of flight. They have limited range, high noise, and translucent material issues but require less processing power than software-based approaches [68].

**Figure 3:**
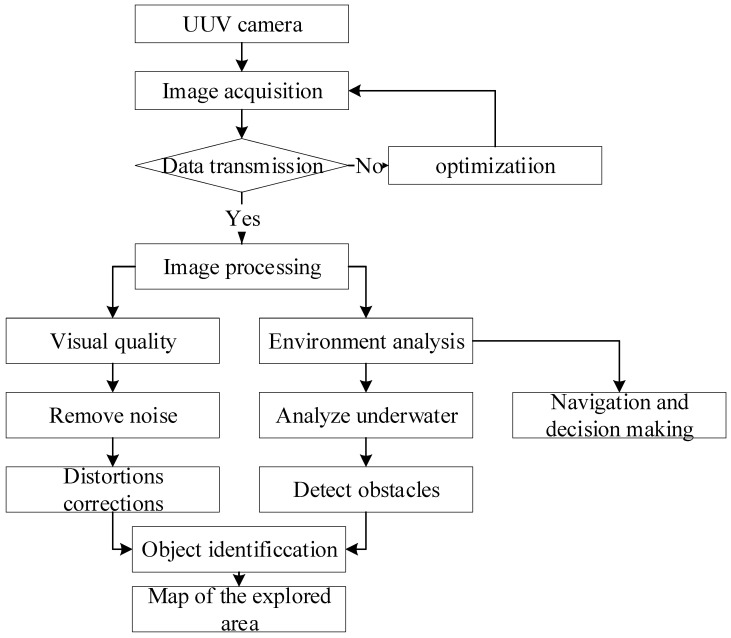
UUV camera image processing flowchart.

Data transmission begins after UUV camera picture acquisition, as in Figure 3. Image processing improves visual clarity, removes noise, and corrects distortions in transmitted data. After processing, the photos are used for environmental analysis, which comprises recognizing impediments, identifying items, and mapping the investigated area. The data are used for navigation and decision making.

Active and passive sonar sensors detect underwater sound waves. Active sonar searches and positions underwater, whereas passive sonar tracks target distances. Water turbidity does not alter single-beam sonar distance information over many meters. For high-resolution 3D mapping, multi-beam sonar measures bottom depth quickly and accurately using many beams. Wrecks and mines are often detected using side-scan sonar, which provides high-resolution seafloor morphology images.

Each sonar type possesses unique functions tailored to particular underwater applications. Active sonar transmits sound pulses to deliver accurate location and distance information, rendering it suitable for extensive searches in diverse aquatic environments. Conversely, passive sonar is undetected as it captures noises from moving objects, rendering it invaluable for covert tracking.

Specialized sonar types are essential: single-beam sonar provides accurate distance readings in murky seas, facilitating long-range detection. Multi-beam sonar excels at three-dimensional mapping by acquiring intricate depth data across vast regions using numerous beams. Side-scan sonar provides high-resolution views of seafloor morphology, facilitating structural analysis for environmental evaluations and danger identification.

**Figure 4:**
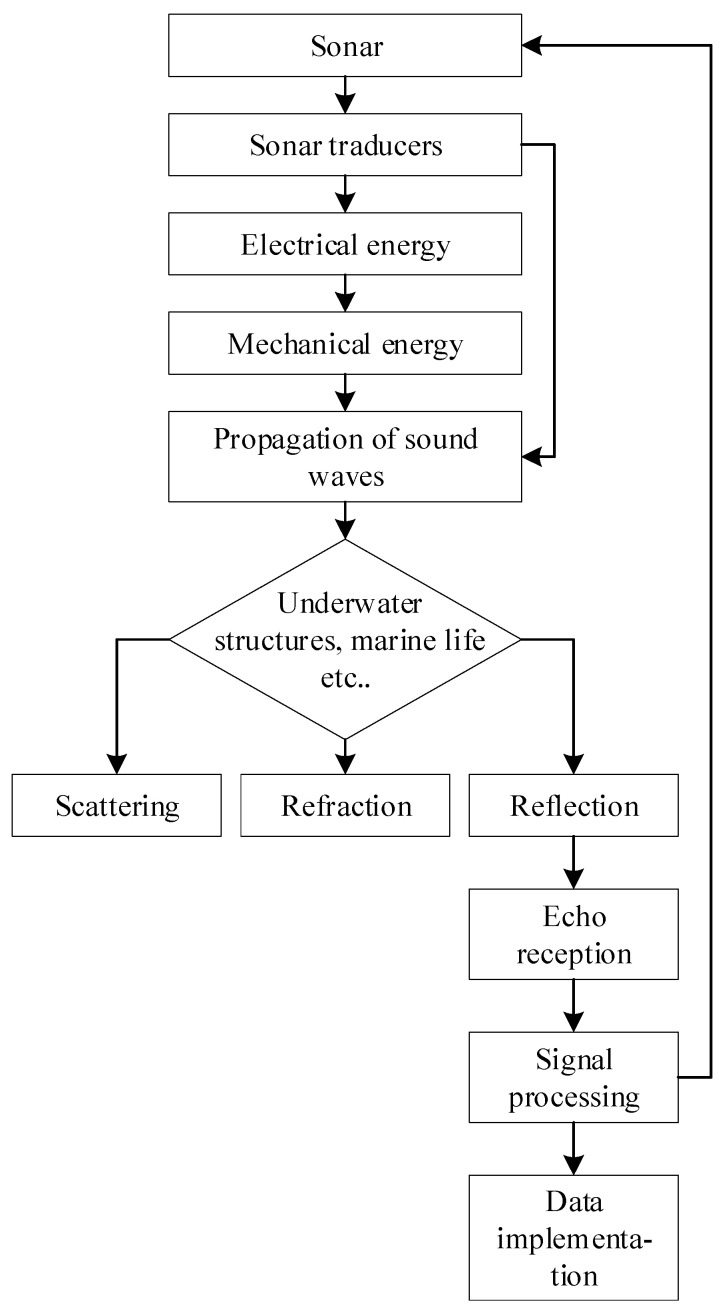
Sonar system in UUV navigation.

Figure 4 depicts the conversion of electrical energy into mechanical energy via sonar transducers, resulting in the transmission of sound waves underwater. The interaction of these waves with subaqueous objects and marine organisms leads to scattering, refraction, and reflection. Reflected waves are subsequently received as echoes, analyzed, and utilized for data implementation, so concluding the sonar operational cycle.

### 2.3. LiDAR Technology

Even in harsh underwater environments, LiDAR sensors provide reliable, high-frequency range data. The high 3D data resolution in texture-limited underwater sceneries includes point cloud data for SLAM systems. LiDAR improves navigational maps by developing 3D models and detecting items on the bottom [69,70].

**Figure 5:**
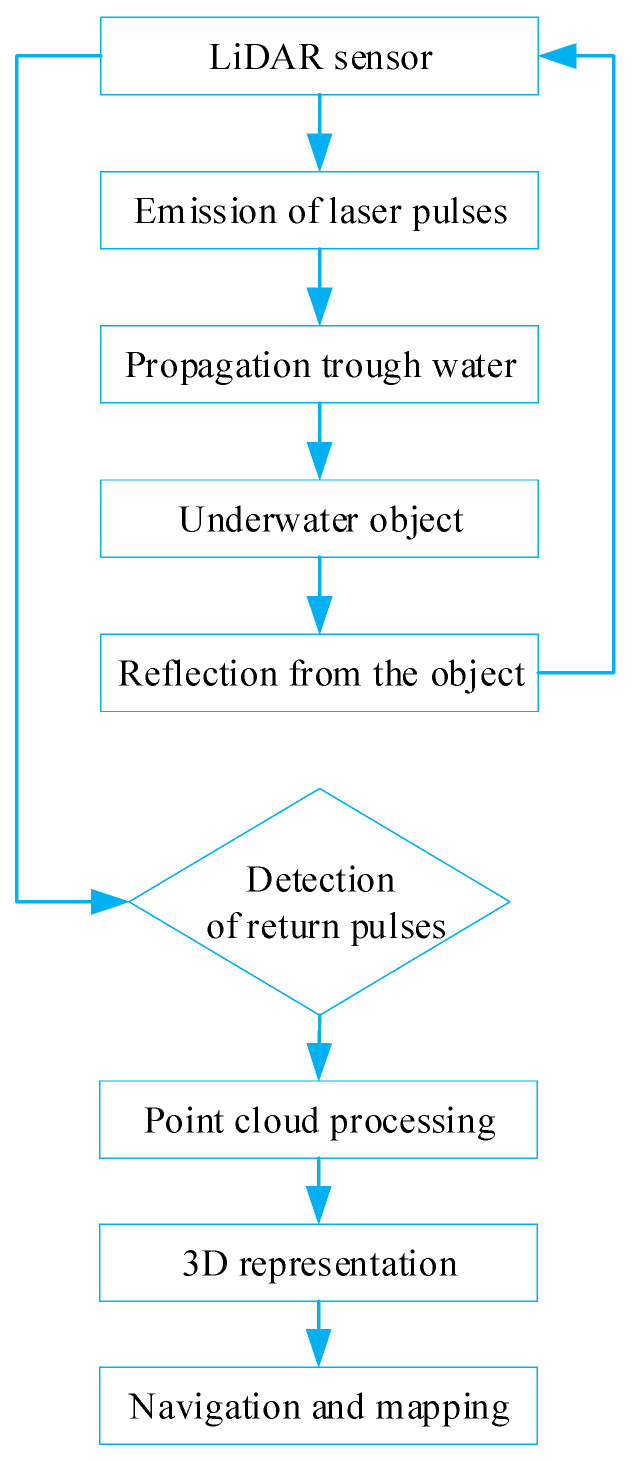
Underwater LiDAR system.

Figure 5 shows the underwater LiDAR system’s sequential operation. Laser systems are emitted, propagate through water, interact with an underwater object, and then are used for navigation and mapping. The LiDAR sensor detects and analyzes reflections to provide a 3D representation and navigational outputs.

**Figure 6:**
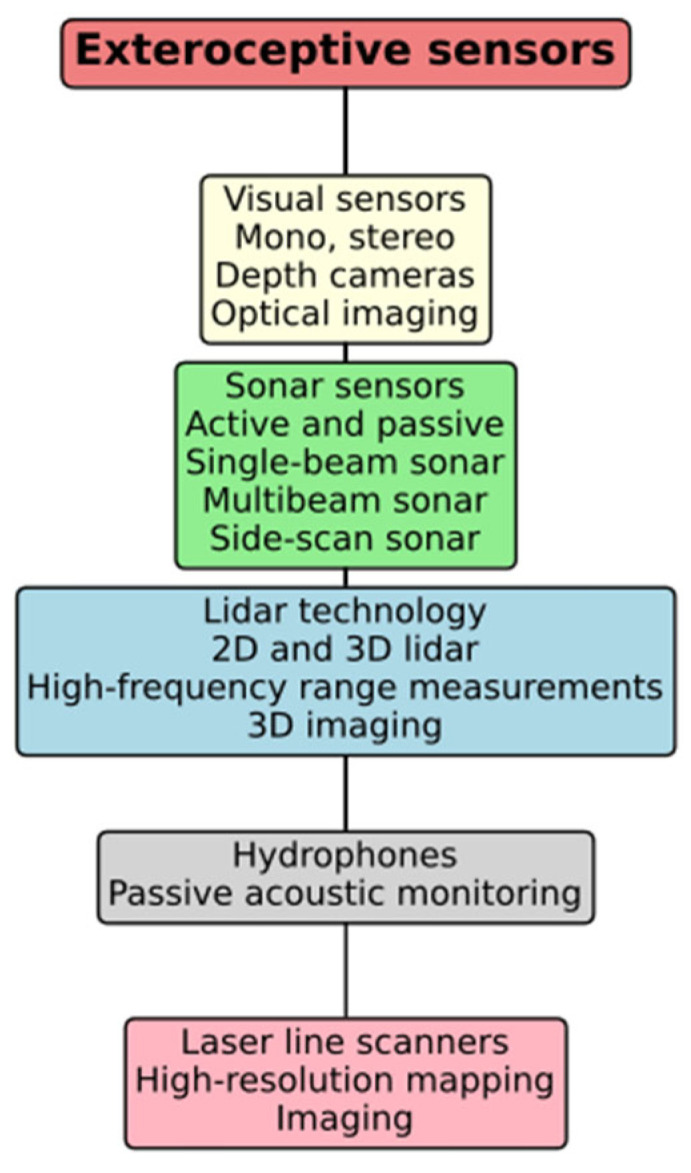
Exteroceptive sensor overview.

This graphic in Figure 6 classifies several types of exteroceptive sensors utilized in underwater settings. Visual sensors, sonar sensors, and LiDAR technology yield several data types, encompassing optical imaging, range measurements, and three-dimensional mapping. Hydrophones facilitate passive acoustic monitoring, whereas laser line scanners assist in high-resolution mapping and imaging. Each sensor type fulfills a distinct function in improving environmental awareness for underwater applications.

The previous section covered underwater SLAM sensors’ types, capabilities, and applications in complex situations. On this basis, the next part discusses how integrating numerous sensors improves SLAM accuracy, resilience, and flexibility in harsh underwater circumstances.

## 3. Multiple Sensor Integration in SLAM Odometry: Strengths and Weaknesses

Numerous scientific investigations indicate that SLAM odometry benefits from incorporating many sensors, providing notable advantages and certain limitations. A significant advantage is the improved durability and precision in a wide range of challenging and intricate settings. Integrating LiDAR with inertial measurement units (IMUs) and optical sensors dramatically enhances the accuracy of mapping and localization, particularly in dynamic environments with fluctuating elements such as pedestrians and cars [71]. In outdoor contexts, incorporating geometric and textural data from LiDAR, IMU, wheel encoder, GPS, and road network data improves localization and mapping accuracy. In interior locations where budget constraints prohibit 3D LiDAR use, a multisensor fusion system employing 2D LiDAR, IMU, and wheel odometry can handle motion degeneracy and geometrically comparable environments, improving robustness and precision [72]. Tightly coupled systems integrating LiDAR, inertial, and visual data use error state iterative Kalman filters and factor graph optimization for real-time error correction and accurate transformation estimate [73]. Multiple LiDAR sensors, including solid-state and spinning LiDARs, improve the robot’s perception by acquiring a high robot ‘station map and accomplishing low-drift ego estimation in featureless interior environments. The complementarity of sensors, such as IMUs’ high-frequency output for rapid handling and cameras’ feature tracking to counter cameras’ drift, makes SLAM systems more robust and able to adapt to unexpected and dynamic surroundings [74].

The complexity and processing effort required to integrate and fuse data from numerous sources are drawbacks of multisensor integration [75]. Using a magnetometer, odometer, and IMU data to improve LiDAR-based SLAM systems in feature-sparse settings requires complex algorithms [76] and factor graph optimization, which can be computationally intensive [77]. Multi-camera [78,79] systems provide rich environmental information and improved feature matching, but dynamic content and changing illumination conditions require advanced techniques like semantic-guided synthetic aperture imaging to maintain accuracy [80]. Multisensor integration [81] in SLAM odometry improves robustness and precision but requires careful computational resources and algorithmic complexity management.

Researchers fuse sensors to improve underwater SLAM systems’ accuracy and robustness. The system method improves underwater SLAM precision and resilience. Vision-Inertial [82], Laser-Vision, and multisensor SLAM are standard approaches [83,84,85]. Multisensor fusion [86,87] has three fusion layers: data, feature, and decision. Although visual SLAM algorithms have improved, they still struggle with low-quality images from rapid camera motions and changing light. Compared to odometers, IMU-assisted sensors improve angular velocity and local location precision, improving SLAM performance cost-effectively. Loosely coupled visual-inertial fusion approaches estimate IMU and camera [88] motions separately and subsequently fuse them, while tightly coupled methods establish motion and observation equations before state estimation.

The data gathered from these sensors are processed and merged through sophisticated sensor fusion techniques, yielding actionable information for underwater SLAM applications. The fusion system integrates data types such as motion, range, 3D mapping [89], visual data, velocity, depth, temperature, and auditory data, guaranteeing precise and dependable navigation in real time. The subsequent session will cover the fundamental fusion techniques employed in underwater multisensor fusion [90].

After discussing the pros and cons of combining many sensors in SLAM odometry, we will examine the fusion methods and computational strategies that enable it. Section 4 analyses Kalman filters, particle filters, graph-based SLAM, and other sensor fusion approaches, including their mathematical foundations, computational trade-offs, and applicability for underwater environments. This foundation allows us to assess how quantum sensors and AI-driven fusion improve SLAM accuracy and resilience in complicated underwater environments.

## 4. Assessing Sensor Fusion Techniques: Efficacy, Intricacy, and Enhancement in Underwater SLAM

### 4.1. Sensor Fusion Methodologies

Sensor fusion integrates input from several sensors to enhance the accuracy and dependability of state estimates in SLAM systems [91]. In a SLAM system, the state denotes the collection of variables that encapsulate the system’s comprehension of its position, orientation, velocity, and environmental map [92]. The states can be classified into two categories: navigation state and map state.

The navigation state encompasses the system’s position, velocity, and orientation—parameters that delineate the vehicle or robot’s location and movement within space. In underwater SLAM, the navigation state encompasses the coordinates of an underwater vehicle, its orientation (often represented as a rotation matrix or quaternion), and its velocity. The navigation state is often compact and updated in real time as the system progresses.

Conversely, the map state encompasses the characteristics or landmarks in the environment that the system has detected. In underwater SLAM, this may pertain to the locations of sonar-detected entities or keyframe orientations. The map state is generally far larger than the navigation state and expands over time as additional characteristics are identified or the system investigates new regions. The intricacy of the map state may fluctuate based on the system and setting; nevertheless, it often necessitates more advanced methodologies for estimation, especially as the map expands.

Sensor fusion methodologies are utilized to ascertain the two states—navigation and mapping—by synthesizing data from diverse sensors, including IMUs (inertial measurement units), sonar, DVLs (Doppler velocity logs), and cameras. The difficulty in sensor fusion is integrating data from several sensors while considering their specific errors, noise, and biases. Four principal sensor fusion methods employed in SLAM systems are Kalman filters (KFs), extended Kalman filters (EKFs), particle filters (PFs), and Graph-SLAM. Each method possesses distinct advantages and disadvantages, contingent upon the characteristics of the system dynamics, the nature of the sensor data, and the surrounding environment.

a. - Kalman Filters

KF are among the most prevalent sensor fusion methodologies, particularly when both the system dynamics and sensor models exhibit linearity, as well as when the noise inside the system is Gaussian [93]. The Kalman filter is frequently employed to assess the navigation state, including position, velocity, and orientation, through the recursive refinement of the state estimate utilizing new sensor data. The KF algorithm functions by forecasting the system’s condition at each time interval and subsequently refining that forecast upon receiving additional data.

The principal benefit of the KF lies in its capacity to integrate predictions with observations in a statistically optimal fashion. In underwater SLAMs, for instance, the Kalman filter may integrate input from sensors such as IMUs, sonar, and DVLs to ascertain the position and velocity of an underwater vehicle [94,95,96]. This is effective when the system adheres to linear dynamics and the measurements are affected by Gaussian noise [40,97,98,99,100].

The KF algorithm operates in two primary phases: prediction and updating.

Prediction: The Kalman filter predicts the state at time t using the previous state $x_{t-1}$ and control input $u_{t}$:

(1) $$x_{t}=F_{t}x_{t-1}+B_{t}u_{t}+w_{t}$$

where

$x_{t}$ is the state at time t;
$F_{t}$ is the state transition matrix;
$B_{t}$ is the control input matrix;
$u_{t}$ is the control input;
$w_{t}$ is the process noise (assumed to be Gaussian).

Update: Upon the availability of new sensor measurements $z_{t}$, the filter revises the anticipated state. The update phase integrates the Kalman gain Kt, which reconciles the forecast with the measurement:

(2) $$K_{t}=P_{t}^{-}H_{t}^{T}(H_{t}P_{t}^{-}H_{t}^{T}+{R)}^{-1}$$

(3) $$x_{t}=x_{t}^{-}+K_{t}(z_{t}-H_{t}x_{t}^{-})$$

where

$K_{t}$ is the Kalman gain;
$P_{t}^{-}$ is the predicted covariance;
$H_{t}$ is the measurement matrix;
R is the measurement noise covariance;
$x_{t}^{-}$ is the predicted state estimate.

This iterative procedure enables the Kalman filter to perpetually enhance the system’s state estimation by utilizing new information.

b. Extended Kalman Filters

The EKF augments the Kalman filter to accommodate non-linear system dynamics and measurement models. Although the KF presumes that both the system and measurement models are linear, the EKF can linearize these models based on the current state estimation [101,102]. The EKF is appropriate for intricate, non-linear systems frequently found in real-world SLAM applications, including underwater or aerial vehicles with dynamic motion models and non-linear sensor readings [103,104].

The EKF operates analogously to the KF, with a principal distinction: it linearizes the system and measurement models utilizing Jacobian matrices at each time increment.

Forecast of state: The state prediction in the EKF adheres to a similar structure as in the KF, while incorporating non-linear system dynamics.

(4) $$x_{t}=f\left(x_{t-1,}u_{t}\right)+w_{t}$$

where $f\left(x_{t-1,}u_{t}\right)$ is the non-linear state transition function.

Jacobian matrices: To linearize the system, the EKF calculates the Jacobian matrices of the state transition and measurement functions.

(5) $$F_{t}=\frac{𝜕f(x_{t-1,}u_{t})}{𝜕x_{t-1}}$$

(6) $$H_{t}=\frac{𝜕{h(x}_{t})}{𝜕x_{t}}$$

where $f_{t}$ and $h_{t}$ are the non-linear state transition and measurement functions, and $F_{t}$ and $H_{t}$ are their Jacobians.

Update: The update phase in the EKF resembles that of the KF but utilizes linearized models.

(7) $$K_{t}=P_{t}^{-}H_{t}^{T}(H_{t}P_{t}^{-}H_{t}^{T}+{R)}^{-1}$$

(8) $$x_{t}=x_{t}^{-}+K_{t}(z_{t}-h\left(x_{t}^{-}\right))$$

Jacobian matrices enable the Extended Kalman filter to manage non-linearities; however, this introduces increased computational complexity and the risk of inferior performance if the linearization inadequately approximates the actual non-linearity.

c. Particle Filters

PFs, or sequential Monte Carlo (SMC) approaches, are an effective technique for predicting the state of systems characterized by non-linear dynamics and non-Gaussian noise. Particle filtering is especially advantageous when the system’s state distribution is multimodal, complicating approximation using a singular Gaussian distribution as utilized in Kalman filtering and extended Kalman filtering [105].

In PFs, the state is shown by a collection of particles, with each particle symbolizing a potential state of the system [106]. The filter operates by advancing the particles through time according to the system’s dynamics and adjusting their weights in response to sensor measurements [107].

Forecast: Each particle is advanced according to the system’s dynamics and control input.

(9) $$x_{t}^{\left(i\right)}=f\left({x_{t-1}^{\left(i\right)}}_{,\;}u_{t}\right)+w_{t}^{\left(i\right)}$$

where $w_{t}^{\left(i\right)}$ is the process noise for particle i.

Update: The weight of each particle is adjusted according to the probability of the measurement relative to the particle’s anticipated state:

(10) $$w_{t}^{\left(i\right)}=w_{t-1}^{\left(i\right)}*p\left(z_{t}\right|x_{t}^{\left(i\right)})$$

where $p\left(z_{t}\right|x_{t}^{\left(i\right)}$) is the likelihood of the measurement $z_{t}$ given the predicted state $x_{t}^{\left(i\right)}$.

After the adjustment of particle weights, the system executes resampling to produce a fresh collection of particles, emphasizing those with elevated weights.

d. Graph-SLAM

Graph-SLAM is a global optimization method for SLAM that constructs a graph of robot poses and observed landmarks. This method utilizes graph vertices to denote the robot’s poses, encompassing its positions and orientations at various time intervals, as well as the landmarks, which are features or observable items inside the environment [108,109,110]. The edges signify the constraints between these postures and landmarks, usually obtained from sensor data (e.g., range, bearing) or odometry.

The objective of Graph-SLAM is to determine the arrangement of poses and landmarks that optimize the likelihood of the observed data while adhering to the limitations [111,112]. This procedure often entails optimizing a nonlinear least-squares cost function, which modifies the postures and landmarks to reduce the discrepancy between predicted and observed sensor values [108,109,113].

The optimization problem is articulated as follows:

(11) $$\underset{x}{\min}\sum_{i,j}||h(x_{i},x_{j})-z_{ij}{\left|\right|}^{2}$$

where

$x_{i},x_{j}$ represent the robot poses at time steps i and j, ${h(x}_{i},x_{j})$ is a function that predicts the relative measurement between two poses, and $z_{ij}$ is the actual measurement between the poses.

The sum is over all pairs of poses i and j that are connected by a measurement constraint.

In Graph-SLAM, the relative measurements $z_{ij}$ are often derived from sensor data (such as laser range finders, sonar, or stereo cameras). The function ${h(x}_{i},x_{j})$ captures the expected measurement based on the relative poses $x_{i}\;\mathrm{and}\;x_{j}$. The optimization aims to reduce the discrepancy between predicted and actual measurements for all pairs of interconnected poses and landmarks.

Graph-SLAM is very advantageous for extensive mapping settings and prolonged robotic operations. It can process substantial volumes of sensor data and measurements, facilitating a comprehensive optimization of the robot’s course and the map. This method necessitates substantial processing resources for optimization, particularly as the number of postures and landmarks increases [114,115,116].

The optimization procedure frequently employs techniques such as Gauss–Newton or Levenberg–Marquardt to iteratively enhance the robot’s trajectory and the environmental map. The algorithm optimizes the poses and landmarks by minimizing the nonlinear least-squares error, iteratively refining the configuration until convergence is achieved.

### 4.2. Comparative Analysis of Sensor Fusion Techniques and Emerging Technologies for Underwater SLAM

**Figure 7:**
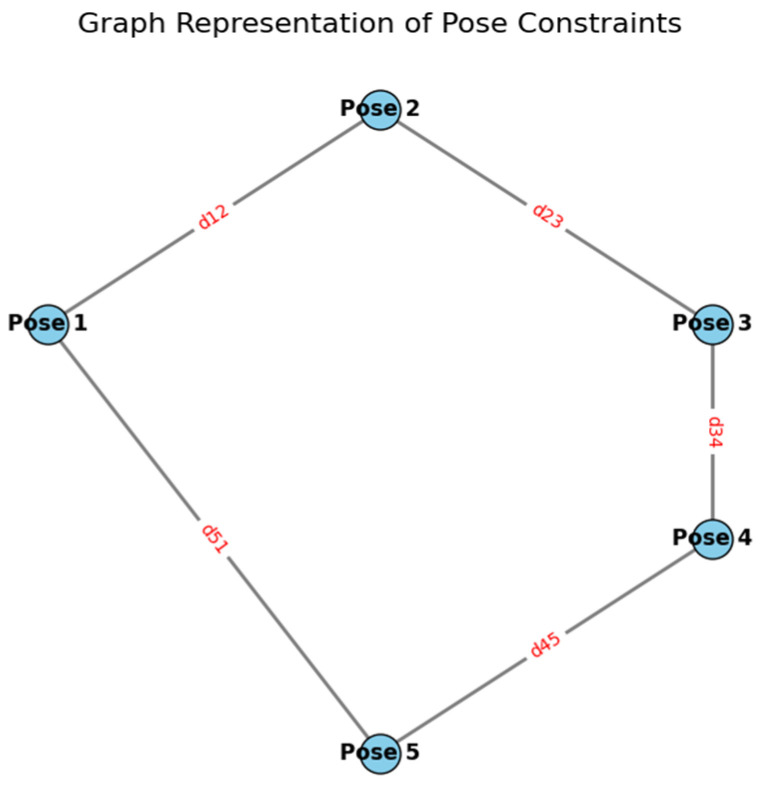
Graph representation of pose constraints.

The graphic in Figure 7 depicts a graph-based [47,48,117,118] SLAM representation, with each node representing a pose of the UUV throughout its course. The edges connecting nodes signify relative pose restrictions obtained from sensor data, like sonar or LiDAR, utilized for localization and mapping [119,120]. The graph structure illustrates how sensor fusion in SLAM amalgamates spatial links between successive UUV points to create an optimized map. These correlations mitigate drift and enhance accuracy in demanding underwater conditions where GPS signals are inaccessible. This visual depiction underscores the essential function of graph-based SLAM in ensuring reliable navigation and precise mapping over extended missions.

a. Complexity analysis and quantitative evaluation

The computing complexity as detailed in Table 1 of each approach varies considerably depending on the environment and the quantity of integrated sensor modalities. The following table delineates the trade-offs among various methods:

b. Quantitative evaluation of sensor fusion techniques

In assessing the practical efficacy of diverse sensor fusion methodologies for underwater SLAM, several critical metrics can be employed to gauge their usefulness, including error rate (RMSE), computational load, and drift over time. These measurements offer insights into the compromises among accuracy, computational complexity, and practical usefulness. The table below consolidates data from many case studies, emphasizing prominent sensor fusion techniques’ comparative advantages and disadvantages, including KF, EKF, particle filters, and graph-based SLAM [129]. Each method is evaluated according to its standard performance in simulated and real-world contexts.

**Figure 8:**
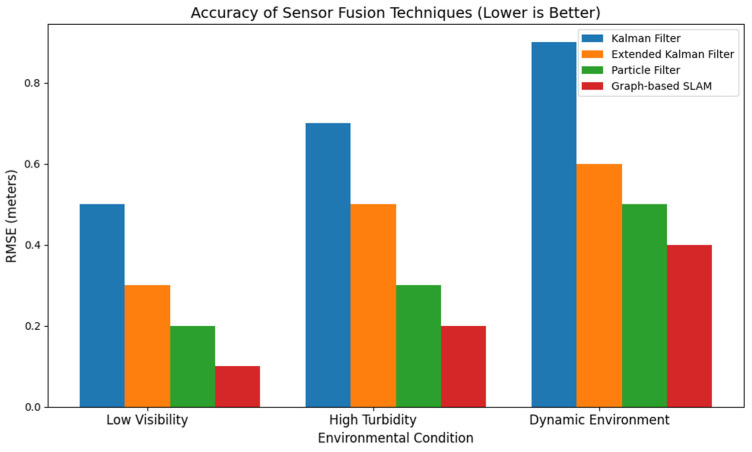
Accuracy of sensor fusion techniques in subaqueous environments.

**Figure 9:**
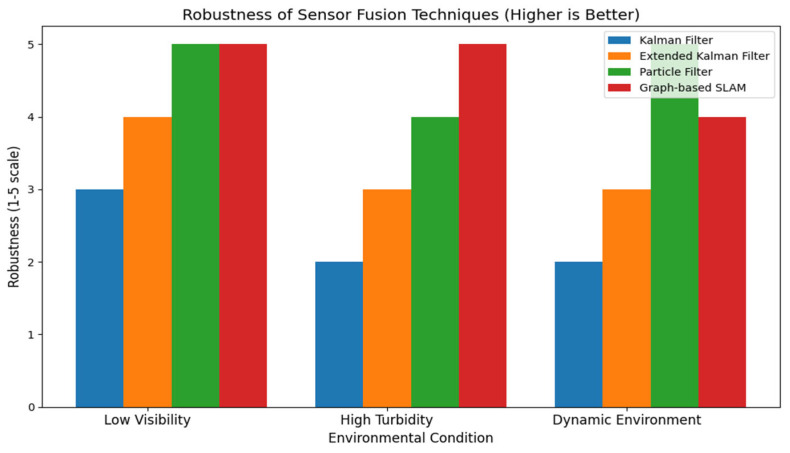
Assessment of the robustness of sensor fusion techniques.

**Figure 10:**
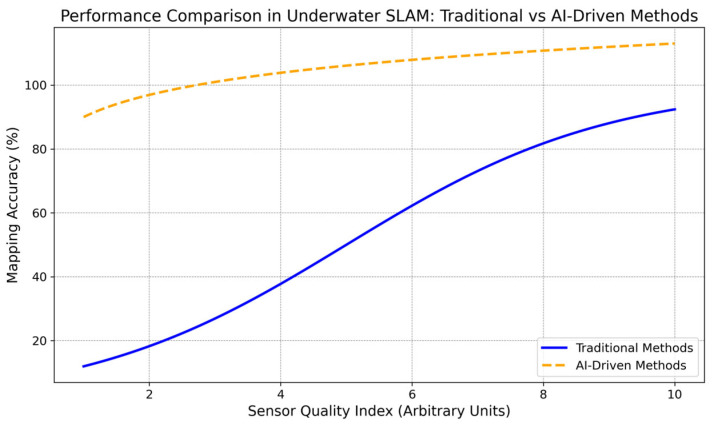
Comparing the performance of AI-driven and conventional sensor fusion techniques for underwater SLAM systems. Not obtained from actual experimental data, this chart is an indicative summary based on a synthesis of literary results.

**Figure 11:**
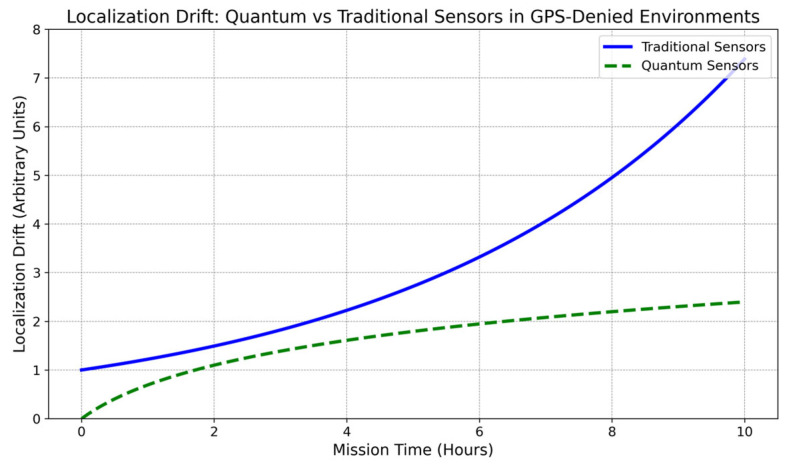
Localization drift in GPS-disabled environments: comparing quantum and conventional sensors. This image is an illustrative summary based on a synthesis of published findings rather than original experimental data.

**Figure 12:**
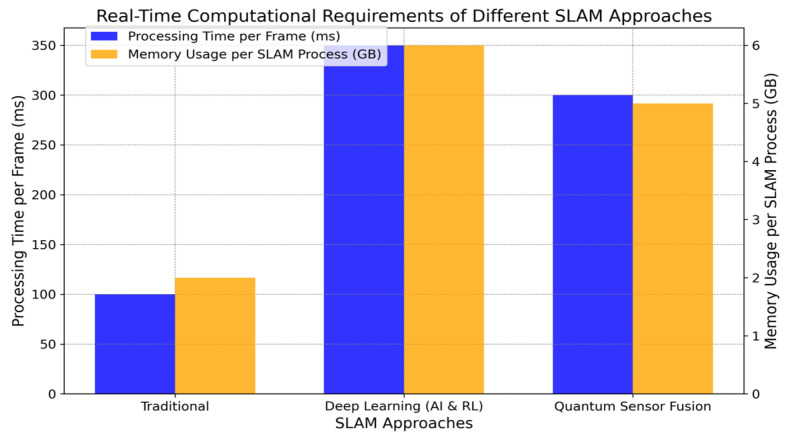
Comparison of the mapping precision of single and multisensor fusion for SLAM systems. This graphic shows a literature synthesis, not experimental data.

Note: Figure 8, Figure 9, Figure 10, Figure 11 and Figure 12 are artistic depictions derived from a synthesis of literature findings. These figures are not based on genuine experimental data or particular calculations. They function as conceptual summaries of overarching trends and attributes documented in prior research, providing visual insight into the relative strengths and weaknesses of diverse sensor fusion methodologies across varying environmental situations.

This graphic in Figure 9 displays a comparative evaluation of the robustness of four sensor fusion approaches. Robustness is evaluated on a scale from 1 to 5, with elevated values indicating enhanced resilience to environmental disruptions, including reduced visibility and turbulence. This image is an example overview synthesized from the literature, not based on real experimental data.

Recent works have examined Kalman filters, particle filters, and graph-based SLAM approaches to handle the unique problems of dynamic underwater settings. Due to environmental factors such as fluctuating currents and turbidity and the lack of GPS, these old methods are limited in adaptability and accuracy. In real-time applications, where quick and adaptable decision making is crucial, these methods are computationally intensive. This figure is a synthesis of literature findings that serve as an illustrative summary and are not derived from original experimental data.

### 4.3. Integration of Quantum Sensors, Deep Learning, and AI-Driven Techniques for Improved Underwater SLAM

Quantum sensors offer unmatched sensitivity to environmental changes, while AI-based methods enable real-time adaptability in data fusion strategies, together providing transformative advantages for reliable and flexible SLAM in complex underwater conditions.

Quantum sensors, which are based on the principles of quantum physics, are extremely sensitive to even minute changes in their surroundings, such as variations in the gravitational and magnetic fields. They are essential for underwater conditions, where typical sensor performance is hindered by turbidity, pressure, and electromagnetic wave attenuation [130]. This sensitivity makes it possible to achieve excellent precision, which is essential for underwater environments. Because of their great stability and minimal drift, they are an important tool for extended missions in places where GPS is not available, which can present substantial issues due to accumulated inaccuracies. Quantum magnetometers, for example, can detect fluctuations in the geomagnetic field. These variations can be used to infer changes in position, therefore providing an alternative navigational cue in situations where traditional signals are not available [131].

Increasing the resilience of SLAM frameworks by integrating quantum sensors is particularly beneficial for situations such as deep-sea exploration, where traditional sensors may not be able to provide accurate results. On the other hand, integrating and miniaturizing quantum sensors within UUVs presents challenges related to power consumption, sensor fusion complexities, and environmental interference [132]. All of these challenges require further development to accommodate practical deployment in real-world scenarios. Moreover, these sensors are being linked with artificial-intelligence-driven filtering algorithms to manage the high-dimensional data that they generate. This allows for the optimization of SLAM processing and accuracy, even in situations that are turbulent and have a limited amount of data [133,134]

Deep learning, especially CNNs, helps process underwater visual and auditory data, addressing issues including picture quality, color distortion, and item dimensions. YOLOv8 and Faster R-CNN accurately identify underwater features for coral reef imaging and AUV operations in low visibility [135,136]. These models improve underwater imaging clarity for biodiversity monitoring and infrastructure inspections by minimizing noise and color distortions.

Sensor fusion improves SLAM systems’ localization and mapping by combining sonar, LiDAR, and camera data with deep learning. Dynamic sensor prioritization—using LiDAR in clear water and sonar in muddy water—improves obstacle recognition, allowing AUVs to navigate complicated terrains without human supervision (Merveille et al., 2024). In complicated underwater environments, deep learning models and sensor fusion improve mapping and localization.

Both supervised and unsupervised deep learning techniques use visual and LiDAR data to improve SLAM. Supervised posture estimation is accurate, but unsupervised methods reduce rotation and translation errors without labeled data [137]. Hierarchical feature encoding and attention techniques improve multi-sensor synthesis pose estimation precision [138]. Laser radar, IMUs, and other data are fused to overcome laser-based SLAM drift with algorithms like EKF for exact environmental mapping. Stable multi-sensor fusion approaches like GNSS simulation provide exact location and velocity data even with malfunctioning sensors.

Deep-learning-based sensor fusion improves underwater mapping by enabling semantic scene understanding in SLAM systems [139]. In dynamic situations, reinforcement learning (RL) improves sensor localization data integration and SLAM stability [140]. Traditional SLAM systems struggle with moving objects, but recent deep learning enhancements identify and compensate for such dynamic aspects. In low-visibility conditions, non-optical sensors like mm Wave radar improve dependability. Due to sensor integration and deep learning problems, underwater SLAM for UUVs needs more research.

IMU, sonar, and LiDAR data processing in real time needs large computational resources, especially in quickly changing surroundings [106,141,142,143]. Distributed computing systems minimize computational load, while GPUs and FPGAs enable parallel processing to reduce latency [144,145,146].

Additionally, dependability issues in UUV operations, such as sensor failures or environmental disruptions, can be mitigated using redundancy and machine-learning-based filtering methods to improve resilience and system efficacy.

The Y-axis in Figure 10 shows the equivalent mapping accuracy (%). In contrast, the X-axis shows the sensor quality. The Y-axis represents equal mapping accuracy (%). In contrast, the sensor quality index (X-axis) displays the sensor system’s ability to collect precise, noise-resistant data (arbitrary units). Traditional methods (solid blue line) enhance mapping accuracy as sensor quality improves, but they cannot reach high accuracy levels. Even with moderate sensor quality, AI-driven approaches (dashed orange line) produce near-optimal mapping accuracy, demonstrating more robust and consistent performance across all sensor quality levels. This shows how AI-driven solutions might improve SLAM in complex underwater environments.

X in Figure 11 represents mission time (hours), and Y represents localization drift (arbitrary units). Traditional sensors (solid blue line) wander exponentially due to error accumulation without GPS correction. Quantum sensors (dashed green line) have a smaller localization drift, allowing them to navigate more accurately in GPS-denied areas during long missions.

Integrating deep learning models with sensor fusion and quantum sensing promotes underwater SLAM by augmenting mapping and localization accuracy in difficult, GPS-denied situations. Recent advancements in model compression, edge computing, and AI-driven filtering enhance the capabilities of autonomous underwater navigation. The subsequent part analyzes experimental data to illustrate the efficacy of these fusion techniques in SLAM applications.

Conventional SLAM techniques, such as KF and particle filters, mitigate drift, noise, and processing constraints but frequently encounter substantial drift and erratic alterations in low-visibility settings [40,104]. Research conducted by Bucci et al. and Techapattaraporn et al. demonstrated the efficacy of Kalman filtering; however, it is deficient in adaptability in dynamic conditions [40,104].

Quantum sensors, as analyzed by Sambataro et al., utilize quantum state discrimination to enhance sensitivity and precision in GPS-denied environments, with quantum sensor networks (QSN) attaining meter-level accuracy, essential for prolonged underwater operations [147,148].

AI-driven methodologies provide real-time adaptability, as demonstrated by researchers by combining noisy and heterogeneous sensor data to improve localization stability in dynamic situations while requiring less processing power than static filters [149]. In contrast to conventional methodologies, AI and deep learning [150] techniques enhance responsiveness and precision in underwater SLAM.

We have examined sensor fusion methods and their performance in underwater SLAM applications. Now, we must consider computing complexity. Real-time SLAM processing demands and optimization solutions for underwater navigation are examined in Section 5. Each fusion method balances processing performance and memory utilization. In resource-constrained situations, hierarchical fusion and distributed processing enable efficient deployment.

## 5. Enhancing Computing Efficiency in Sensor Fusion for Real-Time Underwater SLAM

Sensor fusion enhances underwater SLAM navigation accuracy and resilience, but each technique needs different computational demands that affect processing time and memory. Real-time underwater SLAM requires managing these demands, especially when computational tasks might be shared across UUVs or offloaded to surface stations. Essential sensor fusion approaches’ computation needs and optimization methods for underwater accuracy and real-time performance are discussed in this section.

### 5.1. Kalman Filter Efficiency and Variants

The computationally efficient Kalman filter is appropriate for real-time applications with O(n^2^) complexity scaling, where n is the number of state variables [40,151]. It efficiently processes high-frequency, linear IMU and DVL data streams. State linearization and sigma-point sampling make the EKF and UKF more computationally intensive, but they are suggested for non-linear scenarios [104]. To maintain performance and reduce processing burden, adaptive filtering adapts state space to environmental stability [40]. Further optimization may involve dynamically changing filter types based on environmental complexity, utilizing simpler models for stable settings and advanced filters for difficult ones.

### 5.2. Particle Filter Complexity and Adaptations

The particle filter can handle non-linear, non-Gaussian environments; however, its complexity scaling is O(NM), where N is the particle count and M is the state space dimensionality. Reduced particle numbers simplify processing but may lower dynamic accuracy. GPU-based parallel processing and adaptive resampling, which adjust particle count based on environmental stability, enable real-time particle filters [152]. In stable, resource-constrained underwater environments, event-based sampling reduces duplicate processing by updating particles only when significant environmental changes occur [153,154].

### 5.3. Computational Challenges and Solutions

Real-time applications cannot use particle filters since they are computationally expensive but versatile. With particle count, computing load grows since each particle represents a potential state and requires processing. Recent hardware acceleration and adaptive techniques have helped [155]. Software-only particle filter processing on conventional processors like the Cortex-M1 core takes two orders of magnitude longer than hardware accelerators, making real-time SLAM easier in limited situations [155,156].

Particle weights can be changed to save computation [152]. Particle filters can improve state estimations with exponential weight adjustments in noisy sensor environments. Adaptive weight adjustment and selective resampling keep particles representative of high-likelihood states without using too many particles in computationally limited underwater SLAM applications.

Particle filters are beneficial in non-linear and non-Gaussian conditions, but their computing needs limit real-time applications. However, hardware acceleration and adaptive resampling make them more viable. Particle filters and implementation strategies can increase underwater SLAM performance even in tough conditions. Optimizing particle filter algorithms and hardware implementations may make this resilient technology more accessible and efficient for underwater SLAM applications [157].

### 5.4. Graph-Based SLAM Computational Efficiency and Parallelism

Real-time applications must compute graph-based SLAM. SLAM can be used in real-time on low-power platforms due to data parallelism and GPU acceleration [158]. Collective SLAM systems increase efficiency by dividing computational tasks across multiple players. These frameworks allow low-processing bots to interact and perform SLAM using measurement algebra [159]. This cooperative approach makes SLAM viable for smaller autonomous robots with less computational resources.

Although graph-based SLAM has high mapping precision, computing requirements must be balanced. GPU acceleration, graph sparsification, and multi-sensor fusion minimize computational load but complicate implementation. Integrating dynamic and past information into SLAM enhances mapping accuracy but requires careful data processing to avoid inaccuracy [158]. Further research is needed to optimize graph-based SLAM for real-world applications to balance precision advances with computationally feasible methods.

### 5.5. Deep Learning Models and Computational Considerations

Deep learning for underwater SLAM, specifically feature extraction and object recognition, is popular yet computationally expensive, especially during training [160]. Model reduction, pruning, and quantization minimize these requirements for embedded UUV deployment [161,162]. Deep learning for feature extraction and Kalman filtering for sensor fusion balance computational efficiency and data fusion. Knowledge distillation, where a large model trains a smaller one to perform specific tasks, is another novel strategy that reduces processing overhead without losing accuracy.

### 5.6. Event-Driven and Energy-Efficient Processing

Long-term operations in low-energy underwater environments require energy-efficient processing. Event-driven processing decreases computing load and power consumption in steady situations by processing data only when significant sensor changes occur [163,164]. Neuromorphic computers, which mimic brain-like processing for sensor fusion, conserve energy and speed up SLAM processing [165].

### 5.7. Optimization Strategies for Real-Time Performance

Real-time underwater SLAM in resource-constrained environments involves computational complexity control for accuracy and efficiency. Hierarchical sensor fusion intermittently analyses complex, low-frequency sensors like sonar or cameras and continually incorporates feedback from high-frequency, low-complexity sensors like IMUs to balance accuracy and computing load [166]. Multi-core CPUs and GPUs can reduce latency and enable real-time particle filter and deep learning model execution [167]. In multi-UUV systems, distributed processing decreases UUV workload and enhances system responsiveness by outsourcing computation to surface stations or dividing it between vehicles.

### 5.8. Balancing Accuracy with Computational Efficiency

Sensor fusion is computationally expensive for underwater SLAMs. Particle filters and deep learning models outperform Kalman filtering in complex scenarios but require more computer resources. Hierarchical fusion, parallel processing, and event-driven sampling help SLAM implementations balance these trade-offs and operate in challenging underwater conditions in real time [168].

The graph in Figure 12 contrasts the computing requirements of the four techniques regarding processing load. Reduced values indicate enhanced efficiency, underscoring the real-time relevance of each method in underwater navigation activities. Our subsequent interest is directed to the successful sensor fusion case studies and algorithm implementations in UUV SLAM.

Based on the computational challenges of SLAM sensor fusion, the next section discusses applied case studies of multisensor fusion methods in underwater environments. In Section 6, major experimental results from SLAM investigations in military operations and deep-sea research show how these fusion methods improve localization precision, visibility adaptability, and environmental noise robustness. The assessments show the strengths and weaknesses of different strategies and reveal when certain sensor fusion techniques work well.

## 6. Practical Implementations of Sophisticated Sensor Fusion in Underwater SLAM for Military, Industrial, and Research Endeavors

### 6.1. A Few Case Studies of Successful Sensor Fusion

Military activities, oil and gas development, and deep-sea research require UUVs. Advanced sensor fusion systems integrate sonar, LiDAR, and inertial sensors for covert navigation, enabling underwater surveillance, mine detection, and reconnaissance in GPS-denied areas in the military [169]. In the oil and gas industry, UUVs check pipelines and undersea infrastructure for leaks, even in low visibility. Despite tremendous pressure and low visibility, they construct high-resolution 3D images of unknown locations for deep-sea research [104,170,171,172,173,174].

Incorporating vision-based methodologies, such as feature extraction methods like SIFT, with topological maps improves SLAM efficacy under many environmental conditions, including variations in illumination and noise. This method enhances localization and mapping accuracy, especially in underwater environments where optical and acoustic imaging diverge markedly [175]. Furthermore, feature-based SLAM systems that integrate semantic information enhance robustness by linking feature points to semantic labels, resulting in improved feature-matching precision and loop closure detection [176].

Frameworks such as ORB-SLAM2, when integrated with deep learning for intersection recognition, improve the precision for environmental representations, hence augmenting the overall reliability of SLAM [177]. Incorporating multisensor systems, including LiDAR-inertial odometry and visual-inertial odometry, enhances the resilience of SLAM in intricate situations. Furthermore, real-time location algorithms integrating point-line features with IMU data have demonstrated improved trajectory accuracy and reduced matching errors [178].

Notwithstanding these developments, SLAM systems [179,180] encounter difficulties in dynamic underwater environments. Conventional methods exhibit deficiencies in robustness and accuracy when addressing moving objects; however, contemporary techniques utilizing deep learning and semantic segmentation present potential alternatives. These methodologies emphasize eliminating dynamic feature points while prioritizing static environmental components, enhancing the precision of pose estimation and trajectory mapping. Current research seeks to create algorithms that can manage static and dynamic components in real time, with integrated methods demonstrating the potential for additional error reduction [181]. Integrating deep learning with sensor fusion for underwater SLAM has also shown promising results.

In summary, the selection and integration of appropriate sensor fusion methodologies are pivotal in overcoming the unique challenges posed by underwater environments, where factors such as sensor noise, computational complexity, and variable environmental conditions demand a careful balance between accuracy and efficiency. As advancements in sensor technology and computational techniques continue, these methodologies promise to enhance the accuracy, robustness, and real-time performance of SLAM systems in increasingly complex underwater applications. The following section delves into case studies of successful sensor fusion implementations in underwater SLAM, illustrating the real-world applicability and outcomes of these approaches across various operational environments.

### 6.2. A Few Key Experimental Results from Existing Studies in UUV SLAM

These studies focus on different multisensor fusion approaches, addressing fundamental challenges like visibility, localization precision, and environmental adaptability.

SLAM accuracy depends on underwater picture enhancement and low-visibility feature detection. Liang et al. [168] developed a multisensor system that improves visual quality and contrast by using hybrid attention mechanisms, generative adversarial networks (GANs), and DVL compared to other approaches; the strategy improves feature point detection by 68.18% in MAE and 44.44% in STD. These findings demonstrate the system’s low-visibility AUV performance. Bucci et al. [129] integrated pose-graph SLAM with maximum a posteriori (MAP) estimate using a monocular camera and DVL data. Optimized with an inbuilt reset mechanism to accommodate the computational load, this technique was robust and accurate around Stromboli Island. Li et al. [182] used LiDAR data with EKF in a Rao-Blackwellized Particle Filter (RBPF)-SLAM architecture to improve map clarity and pose accuracy in high drift-risk environments. Techapattaraporn et al. [40] showed that an error-state Kalman filter (ESKF) reduced computing effort by estimating error states instead of full states, improving localization stability. Bucci et al. [104] validated centralized and decentralized UKF techniques in dynamic, low-visibility situations. Both filtering methods are resilient and efficient, essential for underwater navigation.

Underwater SLAM systems using particle filtering efficiently address sensor noise and environmental uncertainty. In their 2021 study, Martínez-Barberá et al. [183] used a sequential Monte Carlo (SMC) architecture with particle filters to manage noise in pipelines using sonar and camera data. Applications demanding great localization reliability benefit from this method. Wang and Qiu [184] used LiDAR and camera data in a multi-modal SLAM framework to achieve 98.9% mapping accuracy and 1.1% error, useful for resource extraction. Vargas et al. [185] used DVL data with visual signals to improve posture prediction and robustness in low-visibility environments, outperforming ORB-SLAM2. Rahman et al. [20] introduced the SVIn2 system, which uses sonar, visual, inertial, and water-pressure data for adaptive, reliable localization in underwater trials. Table 2 provides more details.

### 6.3. Analysis and Limitations of Experimental Results

The reviewed research continuously emphasizes the significance of multisensor fusion in attaining superior localization precision and mapping accuracy in difficult underwater environments. The integration of sensors like DVL, sonar, and cameras alleviates the constraints of single-sensor SLAM methods, improving system adaptability in intricate underwater settings. Nonetheless, specific high-precision fusion techniques, such as particle filters, are computationally intensive, hence constraining their real-time use in resource-limited environments. These trade-offs highlight the necessity for balanced solutions that preserve accuracy while ensuring operational efficiency.

Multisensor fusion methods show promise; however, they frequently use proprietary datasets from controlled contexts, limiting deep-sea application scalability. The computational load of some other advanced algorithms is a major obstacle for real-time applications. Expanding freely available datasets to cover real-world conditions like high turbidity and strong currents would help the community test and assess SLAM algorithms.

### 6.4. Datasets Used in Reviewed Experiments

Most reviews use proprietary or controlled-environment datasets. Public datasets encompassing a range of marine conditions (e.g., high turbidity, deep-sea pressure) are needed to improve benchmarking among multisensor SLAM approaches and encourage field cross-validation and repeatability.

Section 8 synthesizes recent multisensor fusion studies on underwater SLAM, highlighting significant gains in robustness, precision, and adaptability in autonomous underwater vehicles. Innovative advances in image augmentation, probabilistic estimation, Kalman filtering, particle filtering, multi-modal SLAM, and sonar-based applications are presented per theme. Each area highlights visibility, localization, and environmental adaptation improvements, with tables and numbers for a complete picture.

Section 7 discusses how hybrid quantum–AI models, deep learning, and optimized computational frameworks could improve underwater SLAM’s precision and adaptability after reviewing sensor fusion experiments and their effects. These advances aim to let UUVs autonomously explore and map complicated aquatic environments in real time, even without GPS and fluctuating visibility. The next generation of SLAM systems will improve resilience, computing efficiency, and responsiveness by incorporating quantum sensors and AI, enabling disruptive underwater exploration and monitoring applications.

## 7. Future Directions

To overcome underwater obstacles, underwater SLAM systems must integrate quantum sensors and deep learning. These technologies promise to improve underwater SLAM by improving precision, adaptability, and robustness, allowing UUVs to traverse and map complicated aquatic terrain autonomously.

Future SLAM systems may incorporate specialized quantum sensors, such as quantum magnetometers, quantum gravimeters, and atomic clocks, to negotiate underwater obstacles. Atomic clocks provide remarkable timing precision, essential for reducing localization drift over prolonged durations in environments without a GPS. Atomic clocks offer consistent and accurate timing, enabling the synchronization of data from several sensors and minimizing cumulative errors in positioning and trajectory precision [186,187]. Quantum sensors markedly boost the stability of UUV positioning, whereas atomic clocks substantially improve timing precision, a critical factor in complex underwater navigation.

Future SLAM systems may incorporate quantum sensing alongside AI-driven filtering and reinforcement learning (RL) to facilitate real-time flexibility. For instance, UUVs outfitted with quantum magnetometers and artificial intelligence algorithms might adjust to fluctuating underwater conditions, including currents and turbidity, even in the absence of a GPS. In this configuration, quantum sensors would deliver high-precision data, while AI algorithms would dynamically modify SLAM settings according to real-time environmental inputs [147]. This combination would be particularly advantageous for prolonged missions, such as deep-sea exploration and marine ecosystem surveillance.

Deep learning models, including convolutional neural networks (CNNs) and reinforcement learning, enable unmanned underwater vehicles (UUVs) to analyze intricate sensor data and modify simultaneous localization and mapping (SLAM) settings in real-time. Convolutional neural networks (CNNs) facilitate the identification of essential underwater characteristics by unmanned underwater vehicles (UUVs), whereas reinforcement learning (RL) permits the dynamic adjustment of sensor fusion methodologies in response to real-time environmental data [136,188]. This adaptability can improve navigational precision in situations with obstacles and variable visibility.

Future SLAM systems may integrate quantum sensor data with deep learning algorithms to obtain precise environmental assessments and adaptive decision making. Quantum gravimetric data can improve localization precision, but deep learning can aid in semantic mapping and obstacle detection. This combination allows UUVs to identify and react to environmental changes with exceptional precision, enhancing localization accuracy and navigational safety.

Hybrid SLAM models integrating quantum sensing with deep learning necessitate substantial computational resources owing to the intricacy of processing quantum sensor data and AI-driven adaptation. Future SLAM systems must prioritize computational frameworks that enhance processing efficiency while maintaining accuracy. Possible solutions encompass hierarchical sensor fusion, distributed processing inside multi-UUV networks, and the utilization of GPU or neuromorphic processor acceleration to diminish latency and facilitate real-time processing in demanding underwater environments [106,152].

To implement SLAM on resource-limited UUVs, lightweight AI models like MobileNet and SqueezeNet, along with model compression approaches, can support the deployment of quantum-enhanced SLAM. Distributed processing inside UUV networks may enhance operational capabilities by facilitating computational task-sharing in extensive, intricate underwater environments [189].

As SLAM technology advances, comprehensive and varied datasets are crucial for testing hybrid SLAM models. To guarantee thorough testing, researchers might utilize datasets encompassing a variety of underwater settings, ranging from shallow coastal areas to deep-sea regions. Publicly accessible datasets will expedite development and enable cross-validation among universities and sectors, fostering collaboration and resilience in SLAM models [190].

Deep learning models, specifically CNNs and GANs, can augment SLAM by facilitating UUVs in executing semantic mapping and identifying underwater characteristics in real time. These models enable UUVs to categorize seafloor entities such as coral, sand, and rock formations, thereby improving navigation precision and operational safety. The incorporation of deep learning into SLAM enhances contextual awareness, which is beneficial for applications including marine biodiversity monitoring, subsea infrastructure inspection, and archeological research. Nonetheless, lightweight and real-time deep learning models are essential for UUV deployment to guarantee efficiency and responsiveness [191].

## 8. Conclusions

This paper emphasizes significant breakthroughs in sensor fusion for UUV SLAM, highlighting the revolutionary impact of quantum sensors and AI-driven methodologies in tackling intricate underwater issues. This review’s key contributions are (1) a comprehensive examination of quantum sensors, illustrating their capacity to reduce localization drift and improve SLAM accuracy in GPS-denied environments, and (2) an emphasis on AI-driven filtering techniques, which provide real-time adaptability and resilience in dynamic underwater contexts. This paper distinguishes itself from prior assessments that focus on conventional fusion methods by highlighting the distinctive benefits and prospects of emerging technology. Progressing requires addressing obstacles such as computing needs and data integration, as well as enhancing energy efficiency and system reliability, to advance underwater SLAM. Quantum and AI-driven technologies, emphasizing redundancy, failure detection, and adaptive capabilities, offer considerable potential for scalable, efficient, and resilient UUV operations in intricate marine settings.

## Figures and Tables

**Table 1:** Comparative analysis of sensor fusion methods for SLAM: Benefits, drawbacks, and applications.

Method	Advantages	Disadvantages	Computational Complexity	Optimal Application Scenario
KF	Effective for Gaussian noise linear systems	Problems with non-linearities and non-Gaussian noise	O(n^2^)	Low sensor noise, relatively stable settings [22,27,95,116]
EKF	Addresses non-linearities in sensor data	Resource-intensive owing to the computation of the Jacobian	O(n^3^)	Nonlinear environments characterized by moderate dynamics [44]
PF	Effectively functions in non-Gaussian, nonlinear contexts	Necessitates numerous particles; computationally intensive	O(N·M)	Underwater dynamics and uncertainty [45]
Graph-based SLAM	Very accurate large-scale mapping, especially loop closure	CPU-intensive for real-time operation	O(n^3^) to O(n^4^)	Large-scale settings needing precise long-term mapping [47,48]
Hybrid methods	Integrates the advantages of many filters	Augmented complexity and possible overhead	Variable	Environments characterized by fluctuating dynamics and uncertainty [121,122,123,124,125]
Deep learning approaches	Ability to learn complex sensor data representations	Ability to learn complex sensor data representations	Variable	Underwater environments that are complex and data-rich [126,127,128]

**Table 2:** Experimental results from the reviewed literature.

Study	Sensors Used	Environment	Fusion Technique	Key Results
Liang et al. [168]	Camera, DVL	Low visibility	GAN with attention mechanisms	MAE reduced by 68.18%; STD reduced by 44.44%
Bucci et al. [129]	Monocular camera, DVL	Sea trials	MAP with pose-graph SLAM	Comprehensive mapping with enhanced localization precision
Techapattaraporn et al. [40]	INS, DVL	Simulation	ESKF	Outperformed EKF and INS under DVL loss
Martínez-Barberá et al. [183]	Camera, range sensor	Real-world	Particle filter	Improved localization reliability
Wang and Qiu [184]	LiDAR, camera	Real-world	Multi-modal fusion	98.9% accuracy, 1.1% error rate
Rahman et al. [20]	Sonar, visual, inertial, water-pressure	Benchmarks	Keyframe-based SLAM	Robust initialization, loop closure, and relocalization
Vargas et al. [185]	Camera, acoustic sensor	Experimental trials	Acoustic-visual SLAM	Enhanced robustness compared to ORB-SLAM2
Li et al. [182]	LiDAR, EKF	Various	RBPF-SLAM with EKF	Enhanced map delineations, enhanced pose precision
Bucci et al. [104]	Various, UKF	Sea trials	Centralized/decentralized unscented Kalman filter	Improved resilience, reduced measurement impacts

## Data Availability

Not applicable.
